# Forced IFIT-2 expression represses LPS induced TNF-alpha expression at posttranscriptional levels

**DOI:** 10.1186/1471-2172-9-75

**Published:** 2008-12-24

**Authors:** Susanne Berchtold, Birgit Manncke, Juliane Klenk, Julia Geisel, Ingo B Autenrieth, Erwin Bohn

**Affiliations:** 1 Institut für Medizinische Mikrobiologie und Hygiene, Universitätsklinikum Tübingen, Tübingen, Germany

## Abstract

**Background:**

Interferon induced tetratricopeptide repeat protein 2 (IFIT-2, P54) belongs to the type I interferon response genes and is highly induced after stimulation with LPS. The biological function of this protein is so far unclear. Previous studies indicated that IFIT-2 binds to the initiation factor subunit eIF-3c, affects translation initiation and inhibits protein synthesis. The aim of the study was to further characterize the function of IFIT-2.

**Results:**

Stimulation of RAW264.7 macrophages with LPS or IFN-γ leads to the expression of IFIT-2 in a type I interferon dependent manner. By using stably transfected RAW264.7 macrophages overexpressing IFIT-2 we found that IFIT-2 inhibits selectively LPS induced expression of TNF-α, IL-6, and MIP-2 but not of IFIT-1 or EGR-1. In IFIT-2 overexpressing cells TNF-α mRNA expression was lower after LPS stimulation due to reduced mRNA stability. Further experiments suggest that characteristics of the 3'UTR of transcripts discriminate whether IFIT-2 has a strong impact on protein expression or not.

**Conclusion:**

Our data suggest that IFIT-2 may affect selectively LPS induced protein expression probably by regulation at different posttranscriptional levels.

## Background

Lipopolysaccharide (LPS) induces a very complex host response through toll-like receptor (TLR) 4 in macrophages. Among these responses, the production of inflammatory mediators such as tumor necrosis factor (TNF) and interleukin (IL)-6 as well as reactive oxygen species such as nitric oxide (NO) play important roles in protecting the host against bacterial infection. LPS binds to the TLR4/MD2 complex. Ligand binding leads to the recruitment of adaptor molecules such as MyD88 and Trif. MyD88 signals through a complex signaling cascade leading to phosphorylation of the NF-κB inhibitor IκB. IκB is degraded and NF-κB released in the nucleus where it acts as a transcription factor for inflammatory response genes. In addition MyD88 signaling activates MAP kinases such as p38, JNK and ERK which are also involved in this proinflammatory response. Trif-mediated signaling which is elicited by binding of LPS to TLR-4 or dsRNA to TLR-3 leads to the activation of transcription factors such as IRF-3 which binds to ISRE elements and induces IFN-β (for review see [1-3]. IFN-β in turn induces many interferon inducible genes such as interferon-induced tetratricopeptide repeat proteins (IFIT)-1, IFIT-2 but also amplifies the expression of MyD88 dependent genes such as IL-12 p70, IL-6 or TNF-α (for review see [1-3].

Type I interferons have pleiotropic functions [4] including defense against viruses [5,6] and tumor development [4,7]. and they also play a role in immunopathological disorders such as systemic lupus erythematodes (SLE) [8], sepsis [9] or contribute to pathogenesis of bacterial infections [10]. Meanwhile many type I interferon induced genes have been described [11-13], but it is only partially understood how they contribute to effector functions of type I interferons.

IFIT-1 (ISG56, P56) and IFIT-2 (ISG54, P54) are induced in response to type I and type II interferons, dsRNA, LPS, viral [14] and bacterial infections [15] and they are also found in several chronic diseases such as inflammatory bowel disease (IBD) [16] or SLE. Human IFIT-1 and IFIT-2 as well as mouse IFIT-1 and IFIT-2 are linked genes and located either on human chromosome 10 or mouse chromosome 19[17,18]. The encoded IFIT-1 and IFIT-2 proteins are related and contain multiple tetratricopeptide-repeat (TPR) motifs. It was demonstrated that IFIT-1 and IFIT-2 inhibit translation initiation by inhibiting the action of the eIF3 protein complex [19-21]. Human IFIT-1 binds to eIF3e and blocks the ability of eIF3 to stabilize the ternary complex of eIF2, GTP and Met-tRNA. Mouse IFIT-1 and mouse IFIT-2 bind to eIF3c and block the ability of eIF3 to promote formation of the 48 S pre-initiation complex containing the 40 S ribosomal subunit, the ternary complex, eIF4F and mRNA [21]. Nevertheless so far no genes have been described which are actually negatively regulated by IFIT-2. To define genes inhibited by mouse IFIT-2 we generated a stable cell line which constitutively expresses IFIT-2. Our findings suggest that the 3'UTR of certain transcripts determines the selectivity for IFIT-2 mediated inhibition of protein expression in RAW264.7 cells.

## Results

### IFIT-2 selectively inhibits LPS induced protein expression

**Figure 1:**
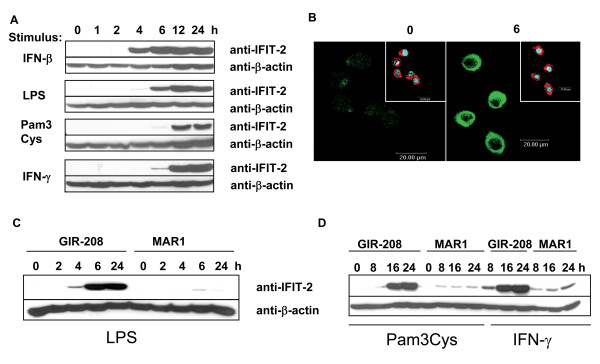
**Protein expression of IFIT-2 in RAW 264**.7 macrophages. RAW 264.7 cells were stimulated with (A) IFN-β (50 U/ml), LPS (1 μg/ml), Pam3Cys (1 μg/ml) or IFN-γ (50 ng/ml), pretreated with GIR-208 or MAR1 prior to (C) LPS, (D) Pam3Cys or IFN-γ stimulation for indicated time periods and IFIT-2 and β-actin expression was detected by immunoblots. (B) RAW 264.7 cells were stimulated with or without IFN-β, staining was performed using anti-IFIT-2 and a secondary Cy2-labeled goat anti-rabbit antibody (green), Phalloidin-Tritc (red) and DAPI (blue) and detected by immunofluorescence. Big pictures; IFIT-2 expression, small pictures overlay of phalloidin and DAPI staining. At least three independent experiments with similar results with exception of B (two experiments).

To confirm previous findings that LPS and interferons induce IFIT-2 protein expression RAW264.7 macrophages were stimulated for different time periods with LPS, IFN-β, Pam3Cys or IFN-γ (Figure 1A). All used stimuli induced IFIT-2 expression but showed differences in the time course of IFIT-2 expression. Thus, while IFN-β induced IFIT-2 expression already 4 hours after stimulation, strong IFIT expression was detectable 6 hours after LPS stimulation and 12 hours after IFN-γ or Pam3Cys stimulation. Immunofluorescence staining of RAW264.7 cells showed that IFIT-2 was located in the cytoplasm (Figure 1B).

Blocking of the IFN-α/β receptor with specific antibodies against the mouse IFN-α/β receptor subunit 1 (MAR1 mAb) demonstrated that LPS, Pam3Cys and IFN-γ induced IFIT-2 expression is dependent on endogenous production of type I interferons (Figure 1C, D). As a control, blocking antibody against the human IFN-γR (GIR-218) was used. This data indicates that IFIT-2 seems to be induced in a specifically type I interferon dependent manner.

### Impact of forced IFIT-2 expression on protein expression

**Figure 2:**
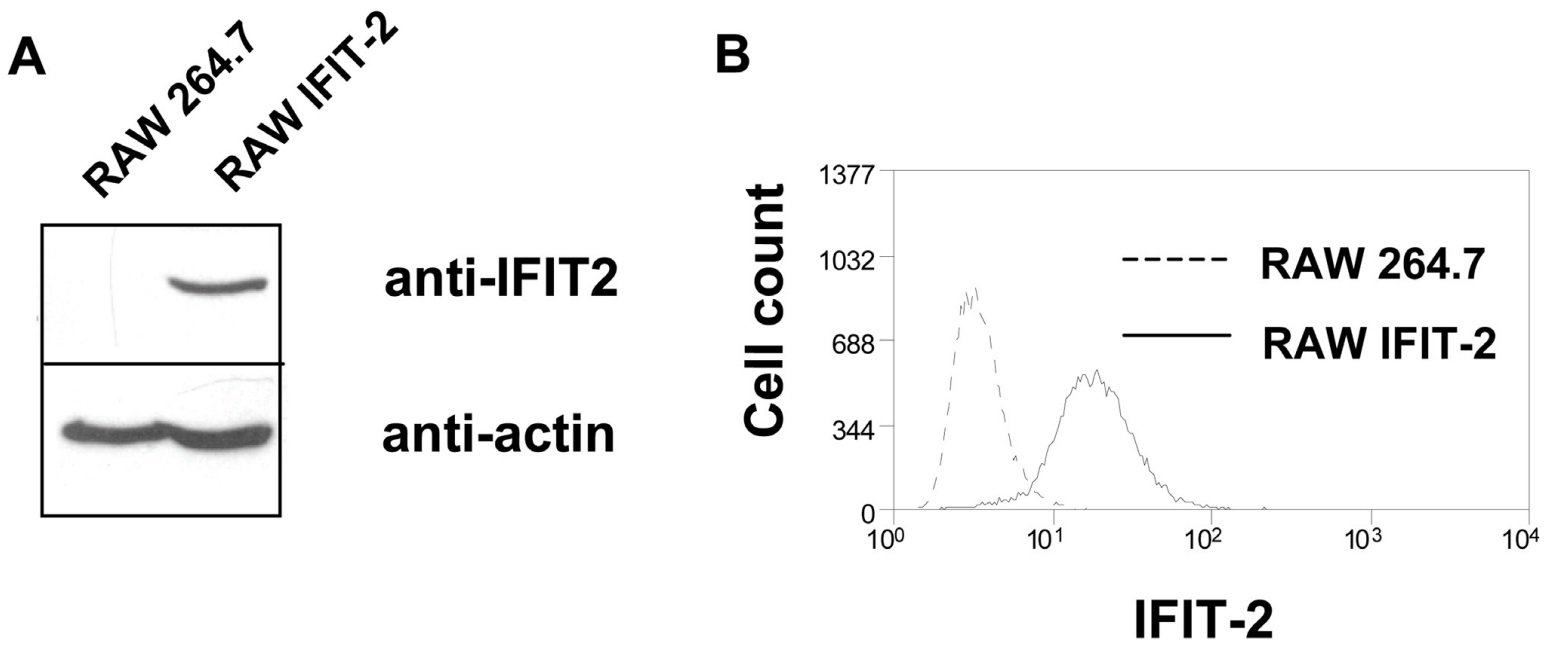
**Protein expression of stably transfected RAW-IFIT-2 cells**. IFIT-2 expression was detected in RAW 264.7 and RAW-IFIT-2 cells by (A) immunoblot or (B) intracellular staining and flow cytometry (at least two experiments).

In previous reports it was demonstrated that IFIT-2 interacts with eIF-3c, interferes with translation initiation and inhibits protein synthesis. So far it was unclear whether IFIT-2 affects protein synthesis globally or in a selective manner. To address this question RAW264.7 macrophages were stably transfected with an IFIT-2 expression vector. The resulting cells (RAW-IFIT-2) were viable and showed no obvious defect in proliferation. Constitutive IFIT-2 expression was detectable (Figure 2).

**Figure 3:**
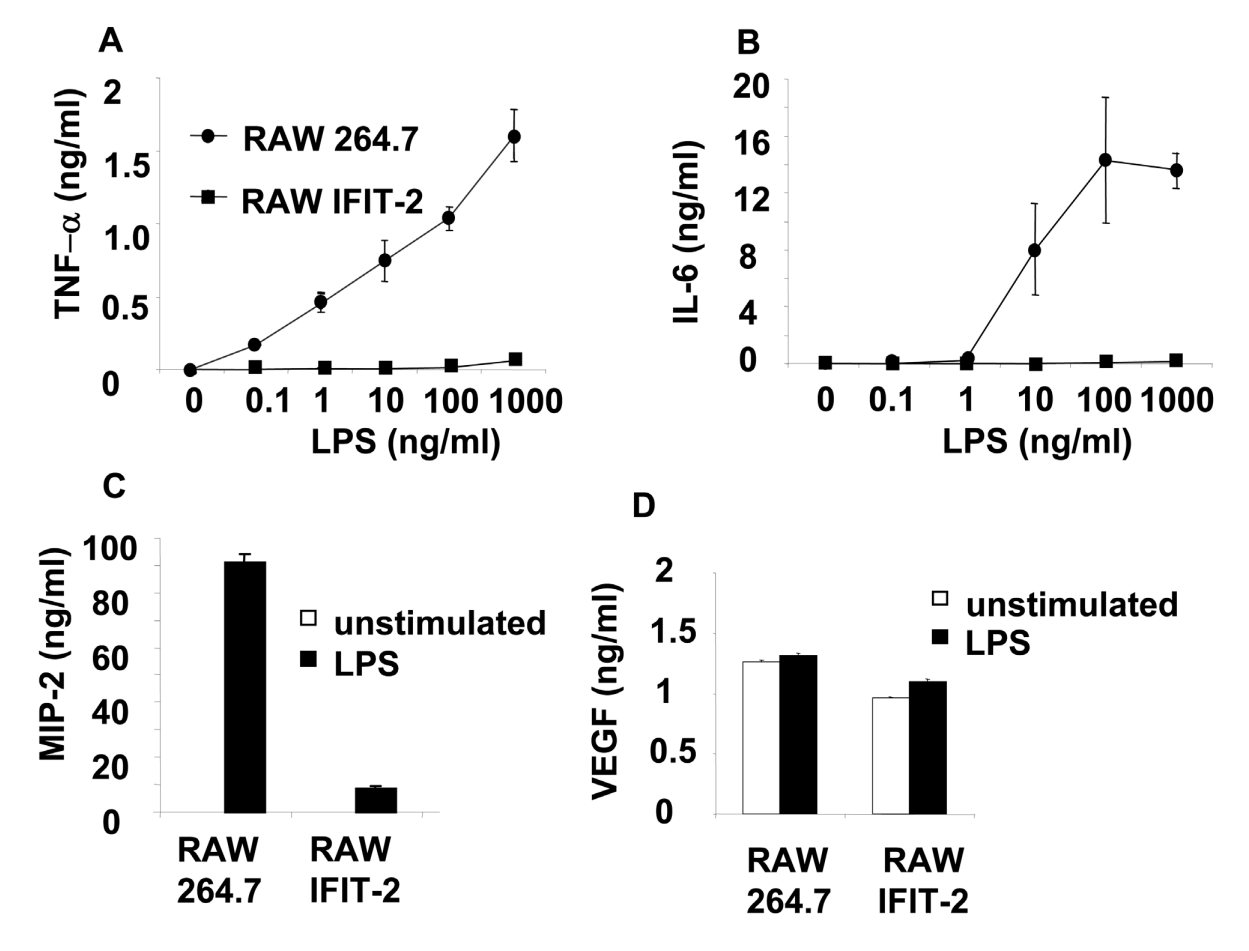
**Forced IFIT-2 expression modulates protein expression**. RAW264.7 and RAW-IFIT-2 cells were stimulated with 1 μg/ml or as stated LPS for 24 h or as stated and (A) TNF-α-, (B) IL-6, (C) MIP-2 or (D) VEGF secretion was determined by ELISA. Significant differences (two-way ANOVA or t-test) were found for TNF-α, IL-6 and MIP-2 (p < 0.001). Three independent experiments with similar results.

To define genes which may be potentially regulated by IFIT-2, RAW264.7 and RAW-IFIT-2 cells were stimulated with LPS and TNF-α, IL-6, MIP-2, and VEGF secretion was determined (Figure 3A–D).

Stimulation of RAW 264.7 macrophages led to a dose-dependent increase of TNF-α secretion. Forced IFIT-2 expression reduced LPS induced TNF-α secretion by more than 90% indicating that IFIT-2 overexpression affects LPS induced TNF-α secretion. TNF-α secretion in RAW 264.7 cells stably transfected with the empty vector pEF4/V5-His A was comparable to RAW 264.7 macrophages after LPS stimulation (data not shown).

**Figure 4:**
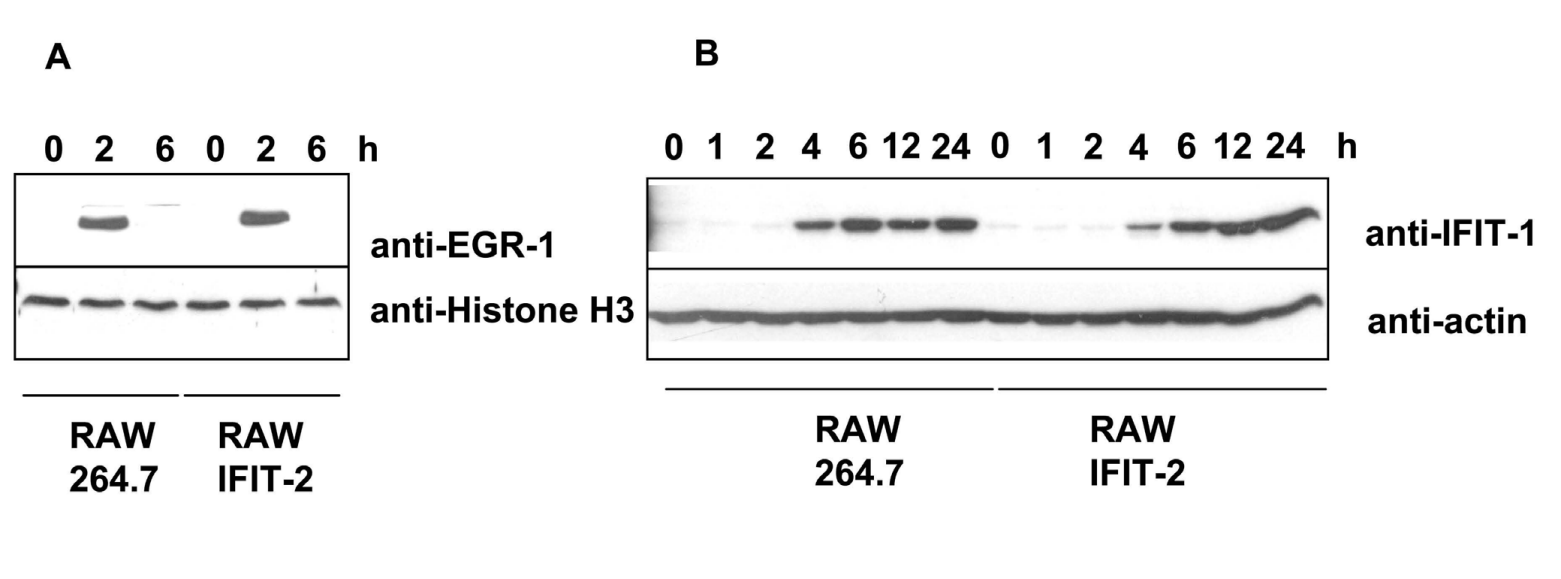
**EGR-1 and IFIT-1 expression in RAW-IFIT-2 cells after LPS stimulation. **Immunoblots were performed after LPS stimulation of cells for detection of (A) EGR-1 and Histone H3 in the nucleus or (B) IFIT-1 and actin in whole cell lysates. Three independent experiments with similar results.

In similar, IFIT-2 overexpression significantly reduced MIP-2 secretion (more than 80%), and IL-6 secretion (more than 95%), as detected by ELISA. In contrast, VEGF secretion was not affected. By using immunoblots the protein expression of several other LPS induced proteins such as IFIT-1 and EGR-1 was determined prior to and after LPS stimulation at different time points. Since EGR-1 expression is predominantly found in the nucleus, protein expression of EGR-1 was determined in nuclear extracts. IFIT-1 and EGR-1 were similarly expressed at indicated time points in IFIT-2 and parental RAW macrophages (Figure 4), which was also confirmed by quantification of the band intensities of immunoblots. Thus, quantification of EGR-1 expression 2 h after LPS stimulation revealed a ratio of EGR-1 found in RAW264.7 compared to RAW-IFIT-2 cells of 0.9 ± 0.2 :1 (n = four experiments).

Taken together, our data indicate that forced IFIT-2 expression affects LPS induced protein expression in a selective manner.

### IFIT-2 does not affect substantially LPS induced signal transduction

**Figure 5:**
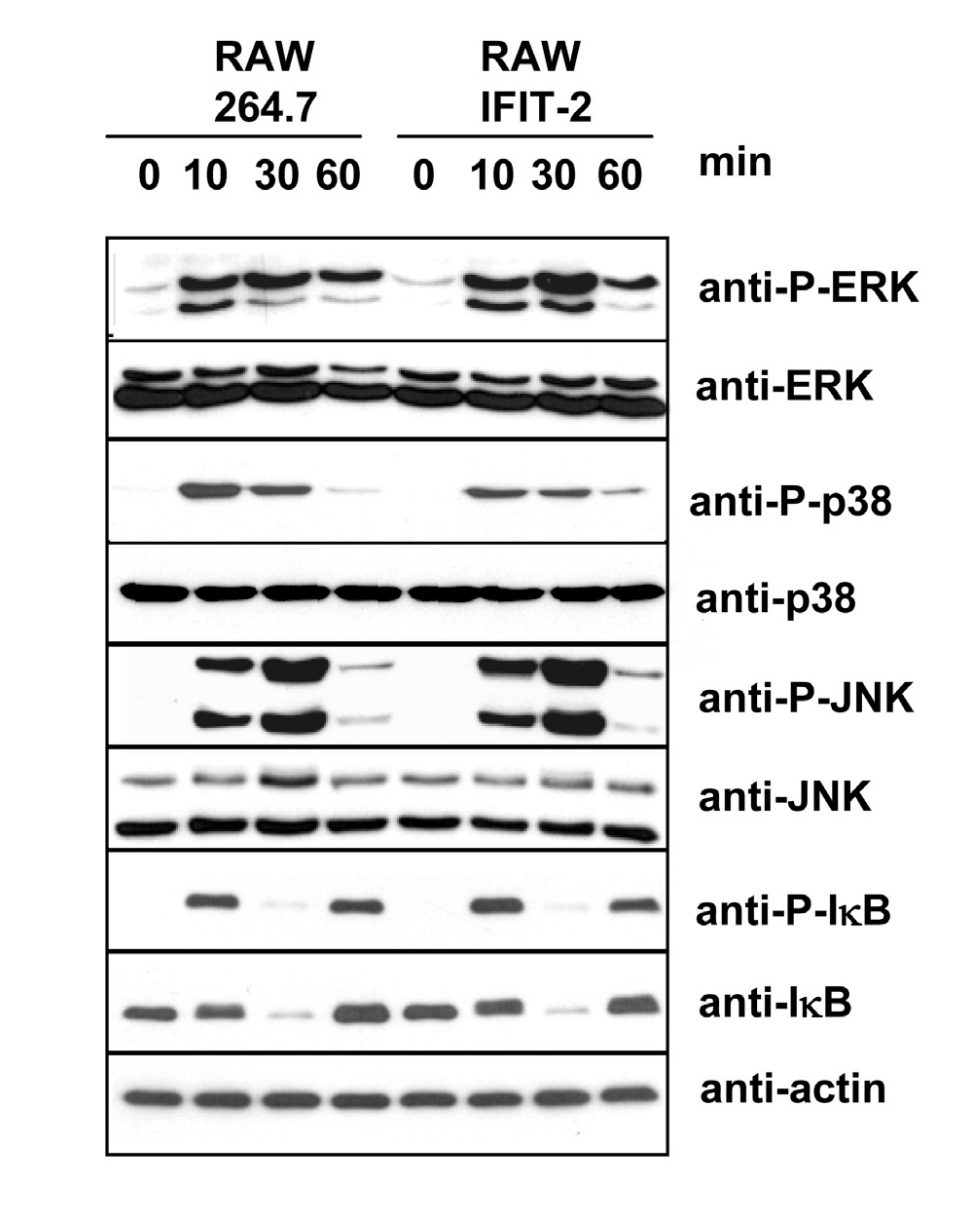
**Impact of IFIT-2 on LPS mediated signal transduction**. RAW and RAW-IFIT-2 cells were stimulated with LPS (1 μg/ml) for indicated time periods. Immunoblots were performed for total and phosphorylated proteins as indicated. Three independent experiments with similar results.

To investigate whether IFIT-2 overexpression also affects LPS mediated signal transduction RAW 264.7 and RAW-IFIT-2 cells were stimulated with LPS for different time periods and immunoblots were performed to determine phosphorylation of ERK, JNK, p38 and IκB using specific antibodies. As a control total amounts of ERK, JNK, p38, IκB as well as actin were determined (Figure 5). Band intensities of three experiments were quantified, normalized to actin and compared for each time point between RAW264.7 and RAW-IFIT2 cells. Slight differences were found for phosphorylated IκB 10, 30 and 60 minutes after LPS stimulation (ratio RAW264.7 : RAW-IFIT-2; 0.7 ± 0.1, 0.7 ± 0.1 and 0.7 ± 0.3) and for phosphorylated p38 only 10 minutes after stimulation (ratio RAW264.7 : RAW-IFIT-2; 1.4 ± 0.1). Taken together, IFIT-2 overexpression did not or did only marginally affect phosphorylation of the investigated signaling molecules. In addition, the expression patterns of the total amounts of these proteins over time after LPS stimulation showed only small variations (data not shown). These data suggest that IFIT-2 overexpression does not affect substantially LPS mediated signal transduction.

### IFIT-2 decreases LPS induced TNF-α mRNA expression by affecting mRNA stability

**Figure 6:**
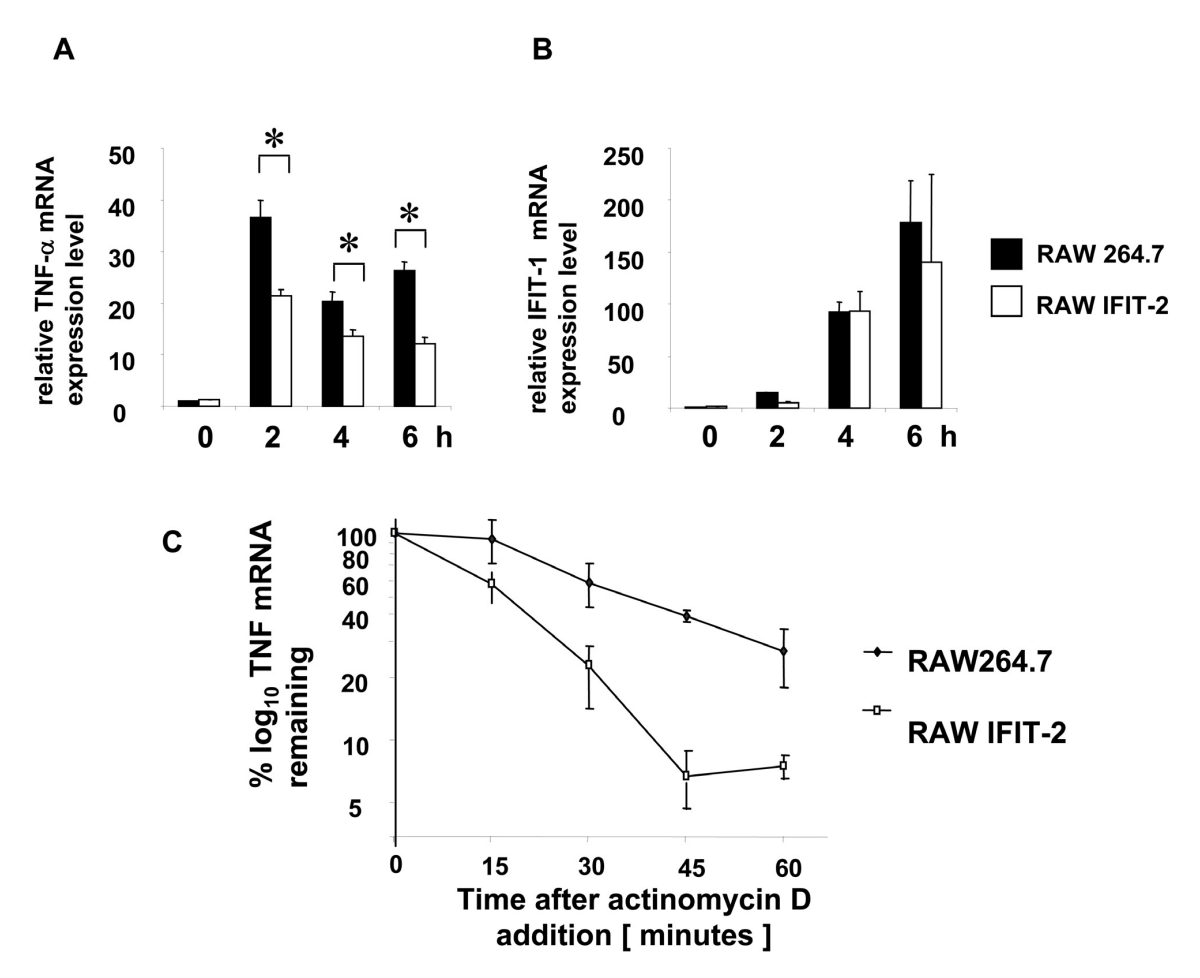
**TNF-α and IFIT-1 mRNA expression in RAW-IFIT-2 cells**. RAW and RAW-IFIT-2 cells were stimulated with LPS (1 μg/ml) for indicated time periods and mRNA expression was detected for (A) TNF-α and (B) IFIT-1 by Real Time RT-PCR normalized to the house keeping gene RPL8. Data are shown as fold increased expression compared to unstimulated cells. Asterisks indicate significant differences p < 0.005 (Two-Way Anova). (C) Cells were stimulated for two hours with LPS and then treated for indicated time periods with actinomycin D. TNF-α and RPL8 mRNA expression was determined by Real-time RT PCR. Data are shown as percentage of the remaining mRNA after actinomycin on logarithmic scale. T_1/2 _was determined using the means and standard deviations summarizing three independent experiments.

If IFIT-2 only affected protein expression, LPS induced TNF-α mRNA expression would be expected to be similar in RAW and RAW-IFIT-2 cells. To investigate whether mRNA expression is affected by IFIT-2, RAW264.7 and RAW-IFIT-2 cells were stimulated and IFIT-1 and TNF-α mRNA expression was determined by Real-time RT-PCR (Figure 6A–B). While LPS induced IFIT-1 mRNA expression was not affected by IFIT-2 overexpression, TNF-α mRNA expression levels were decreased significantly up to two-fold four and six hours after stimulation.

To assess whether mRNA stability was affected by IFIT-2 overexpression, RAW264.7 and RAW-IFIT-2 clones were stimulated with LPS for 2 hours. Subsequently cells were treated with actinomycin D and mRNA expression was determined by Real-time PCR at different time points (Figure 6C). The stability of TNF-α mRNA was significantly decreased in RAW IFIT-2 cells. The half-life of TNF-α mRNA in LPS stimulated RAW264.7 cells (t_1/2 _= 25 minutes) was approximately two-fold higher than in RAW-IFIT-2 cells (t_1/2 _= 13 minutes). This finding explains the differences in TNF-α mRNA levels found between LPS stimulated RAW264.7 and RAW-IFIT-2 cells but not the differences in protein expression levels.

Our data indicate that forced IFIT-2 expression selectively affects mRNA stability and protein expression of some genes, e.g. TNF-α. This raised the question by which mechanism IFIT-2 specifically affects protein expression.

### Characteristics of the 3'UTR of transcripts specify IFIT-2 mediated control of protein expression

**Figure 7:**
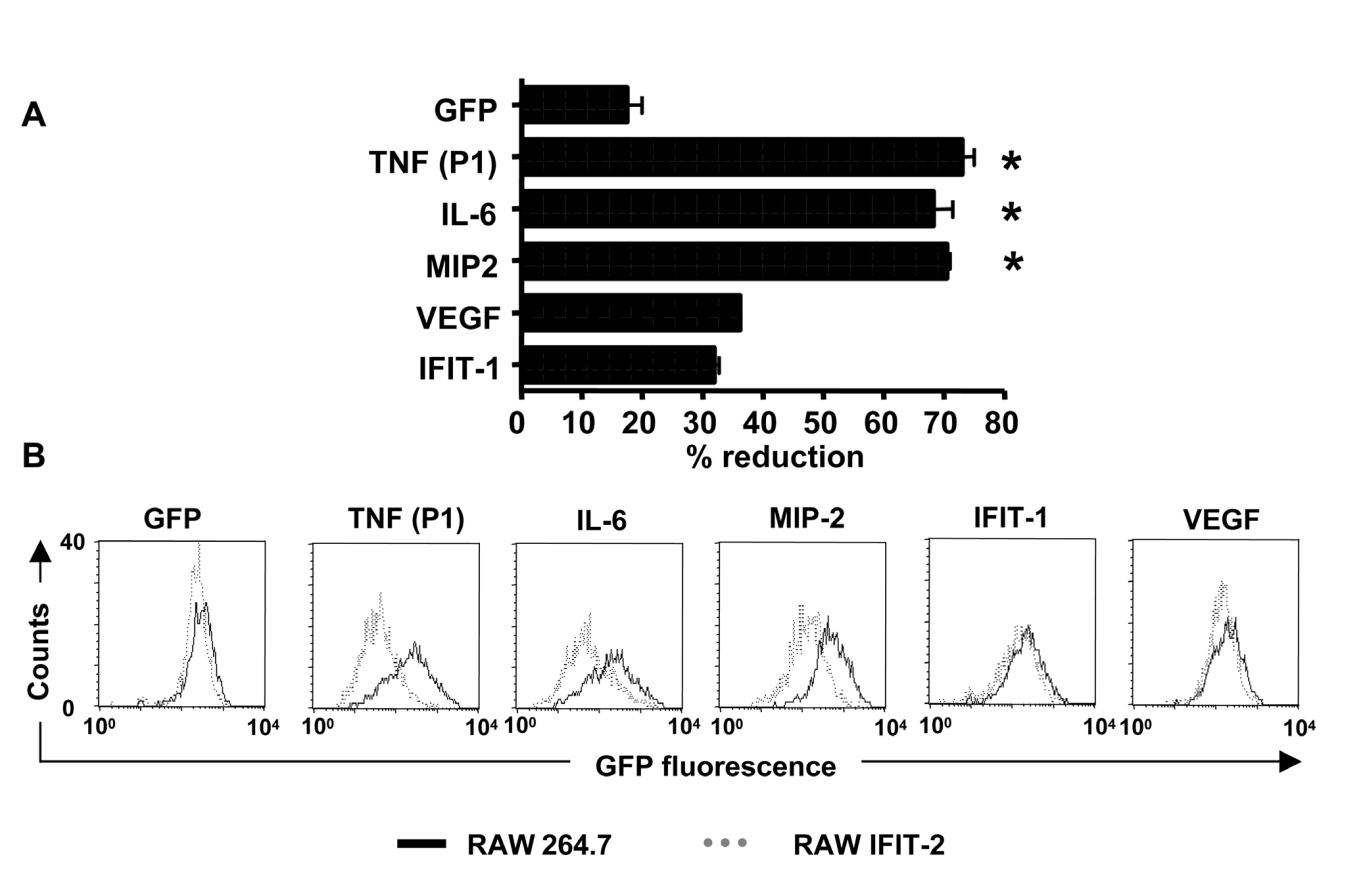
**Impact of 3'UTRs on IFIT-2 mediated inhibition of protein expression**. RAW and RAW-IFIT-2 cells were co-transfected with expression vectors encoding GFP with no 3 'UTR (GFP) or GFP with the indicated 3'UTRs (TNF, IL-6, MIP-2, VEGF, IFIT-1) and an expression vector encoding cherry with no 3'UTR for four hours. Flow cytometry of cells was performed and gated on cherry expressing cells representing efficiently transfected cells. (A) shows the mean of several experiments (n = 3–8) of the percentage of reduction of the mean GFP fluorescence of RAW-IFIT-2 compared to RAW264.7 cells for each construct used. (B) illustrates the histograms of the GFP fluorescence of one representative experiment. Asterisks indicate significant differences (p < 0.05, one-way ANOVA, Dunnett test) compared to control (GFP, no UTR).

Previous studies showed that distinct elements in the 3'UTR such as the GAIT element which was found in the 3'UTR of ceruloplasmin play an important role for translational control [22-24]. Therefore we hypothesized that the 3' UTR of transcripts might determine the selectivity of IFIT-2 to inhibit protein synthesis. To investigate whether characteristics of different 3'UTRs may be important for IFIT-2 mediated inhibition of protein expression, RAW 264.7 and RAW-IFIT-2 cells were co-transfected with an expression vector expressing cherry flanked by a polyA signal but without 3'UTR (as a control for transfection efficiency) and different expression vectors encoding for EGFP flanked by no 3'UTR (GFP), the TNF-α, MIP-2, IL-6, IFIT-1 or VEGF 3'UTR. Subsequently cherry and EGFP expression was determined by flow cytometry (Figure 7). To ensure that only transfected cells were investigated, only cells were analyzed which expressed cherry. The mean fluorescence for EGFP expression was comparable in RAW264.7 and RAW-IFIT-2 cells if an EGFP expression vector was used flanked by no 3'UTR. Transfection experiments using EGFP constructs flanked by the 3'UTR of TNF-α, IL-6 and MIP-2 revealed that EGFP expression was approximately 70% reduced in RAW-IFIT-2 compared to RAW264.7 cells. In contrast the 3'UTRs of IFIT-1 and VEGF had only a minor effect on EGFP expression. These data indicate that the 3'UTR plays an important role for the specificity of IFIT-2 to inhibit protein expression in RAW macrophages. One of the best studied 3'UTRs is the TNF-α 3'UTR and regions are defined which are important for posttranscriptional regulation such as the AU-rich region (ARE) which controls mRNA decay and translation and the constitutive decay element (CDE) which also affects mRNA stability [25-27].

**Figure 8:**
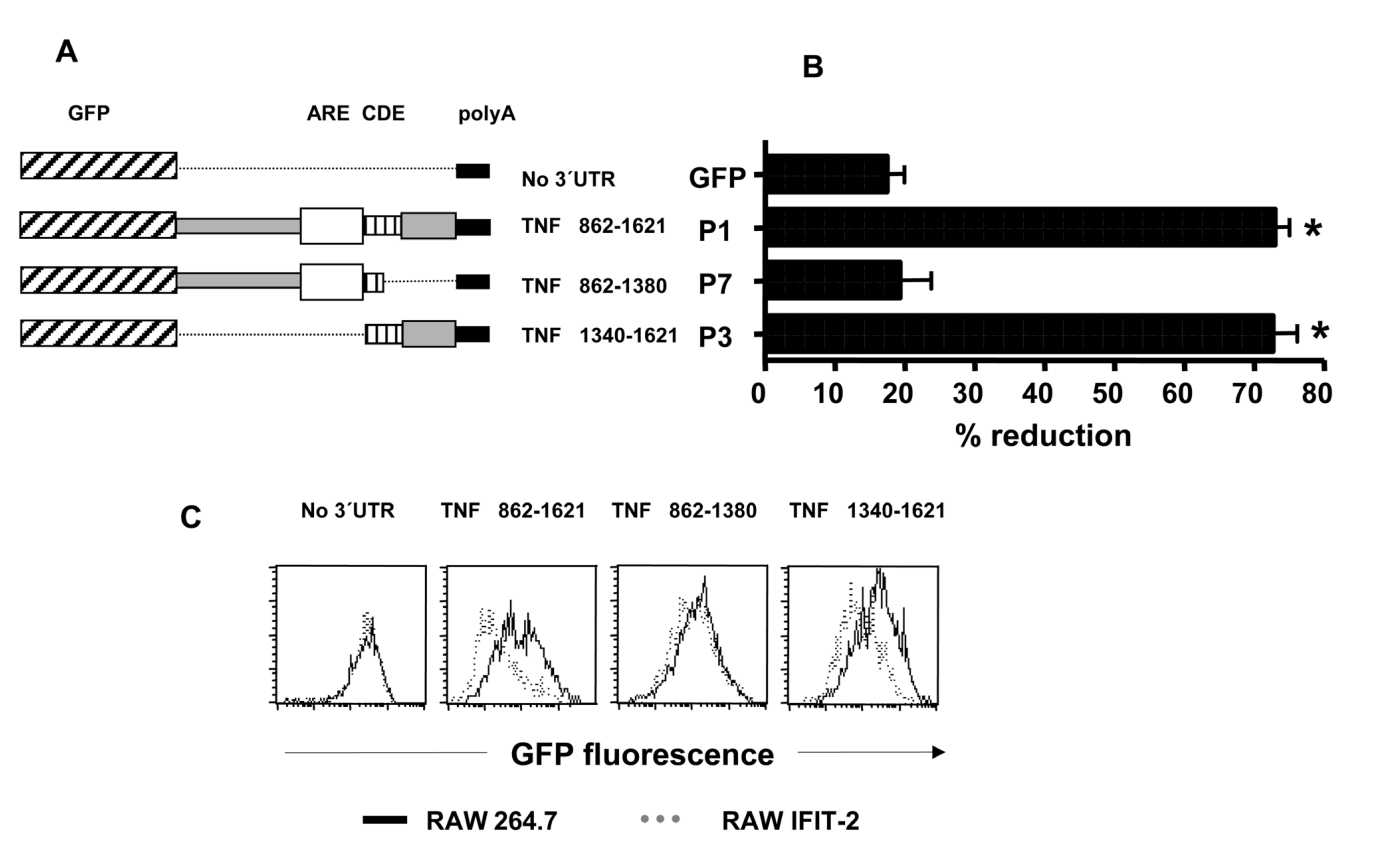
**Impact of deletions in the TNF-α 3'UTR on IFIT-2 mediated inhibition of protein expression**. RAW and RAW-IFIT-2 cells were co-transfected with expression vectors encoding GFP with no 3'UTR (GFP) or GFP with the indicated 3'UTRs and an expression vector encoding cherry with no 3'UTR for four hours. (A) depicts the constructs used, (B) shows the mean of several experiments (n = 3–8) of the percentage of reduction of the mean GFP fluorescence of RAW-IFIT-2 compared to RAW264.7 cells for each construct used. Asterisks indicate significant differences (p < 0.05, one-way ANOVA, Dunnett test) compared to control (GFP, no UTR). (C) illustrates the histograms of the GFP fluorescence of one representative experiment.

To define a distinct element in the 3'UTR of TNF-α, RAW264.7 and RAW-IFIT-2 cells were transfected with constructs encoding EGFP flanked by deletion mutants of the 3'UTR of TNF-α (Figure 8). EGFP expression was significantly reduced by more than 70% in IFIT-2 cells compared to RAW264.7 cells using the constructs TNF 862–1621 (P1) and TNF 1340–1621 (P3). Since P3 lacks the proximal part of the 3'UTR including the AU-rich region one can conclude that the ARE plays no role for the IFIT-2 mediated inhibition of expression. In contrast, transfection with TNF 862–1380 (P7) resulted in a similar GFP expression in both RAW264.7 and RAW-IFIT-2 cells indicating that the distal part of the 3'UTR containing the CDE element is essential for IFIT-2 mediated inhibition of expression.

## Discussion and conclusion

Previous studies demonstrated that IFIT-2 is highly expressed after stimulation with LPS as well as interferons. Our findings confirm these studies and show that LPS as well as IFN-γ induced IFIT-2 expression is dependent on endogenous secretion of type I interferons. Therefore one can assume that IFIT-2 may represent an effector protein which may be responsible for some of the effects which are associated with type I interferon function.

Although IFIT-2 has been known for quite a long time and is strongly expressed in many diseases such as SLE, inflammatory bowel diseases, and viral or bacterial infections, little is known about the biological function of this protein. Recently it was demonstrated that mIFIT-2 inhibits translation by binding to eIF-3c, interferes with translation initiation and inhibits protein synthesis at least in in vitro translation assays [19,21]. Although these studies clearly showed that IFIT-2 could be involved in regulation of protein synthesis it was so far totally unclear whether IFIT-2 affects protein expression in a global or selective manner.

The presented study may shed some light on the function of IFIT-2. As shown here forced IFIT-2 expression does not globally affect protein expression but acts in a more specific manner. We could identify several proteins which are induced by LPS and which are inhibited in protein expression by forced IFIT-2 expression. Examination of LPS triggered phosphorylation of signal molecules of TLR-4 signaling pathways in cells with or without forced IFIT-2 expression excludes that IFIT-2 substantially affects signal transduction. This data indicates that the principal mechanism of IFIT-2 mediated inhibition of LPS triggered cytokine secretion is for instance different to the negative regulation mediated by FLN29 [28]. FLN29 which is induced eight hours after LPS stimulation in a Trif-dependent manner interacts with TRAF6 and inhibits IκB and ERK phosphorylation after restimulation with LPS. Thus, our data using cells overexpressing IFIT-2 argue against the possibility that LPS induced IFIT-2 may affect prominently signal transduction of the tested pathways as it was demonstrated for FLN29 [28]. As shown for TNF-α as an example, forced IFIT-2 overexpression in RAW cells affects to some extent mRNA expression. Actinomycin D treatment shows that reduced TNF-α mRNA expression is associated with reduced mRNA stability. This data strongly argues that IFIT-2 overexpression mediates reduced mRNA stability and therefore acts posttranscriptionally. Nevertheless, we cannot totally rule out that to some extent also transcription might be affected. Since TNF-α mRNA expression is only reduced up to 50%, reduced mRNA stability does not totally explain reduced protein expression. Therefore, we conclude that forced IFIT-2 expression may act at different levels of posttranscriptional regulation. At this point we do not know whether these processes act separately or are linked together. Since not all proteins and especially not all LPS induced proteins are affected by forced IFIT-2 expression we initiated studies to determine possible mechanisms for the action of IFIT-2.

Several other reports pointed out that specific translational control is determined by distinct elements in the 3'UTR to which proteins bind and this interaction of RNA binding proteins with distinct sequence stretches mediates translational silencing. For instance it was described that IFN-γ stimulation leads to formation of the GAIT protein complex consisting of four proteins which bind to distinct stem loops in the 3'UTR of ceruloplasmin [22-24]. These interactions interfere with translation initiation. Therefore we wanted to investigate whether the characteristics of 3'UTRs of the genes we addressed in this study discriminate whether IFIT-2 mediates inhibition of protein expression or not.

By transfection of plasmids expressing EGFP flanked by different 3'UTRs in RAW264.7 and RAW-IFIT-2 cells we could demonstrate that IFIT-2 affects EGFP expression dependent on the 3'UTR attached to the coding sequence of EGFP. Consistent with decreased TNF-α, MIP-2 and IL-6 secretion after LPS stimulation in RAW-IFIT-2 cells, 3'UTRs of TNF-α, MIP-2 and IL-6 mediated lower EGFP expression in RAW-IFIT-2 cells compared to RAW264.7 cells. In contrast, the 3'UTRs of IFIT-1 or VEGF did not influence EGFP expression in RAW-IFIT-2 cells. We conclude that specific properties of the 3'UTRs determine whether IFIT-2 affects protein expression of a certain gene or not. There is already detailed knowledge about different elements of the TNF-3'UTR for the posttranscriptional regulation. Thus, the AU-rich elements (ARE) are important for posttranscriptional regulation. When inserted into reporter constructs, the ARE of TNF-α induces processive deadenylation of the mRNA and efficiently targets the body of the transcript for rapid degradation [26,29]. In addition, the ARE of TNF-α inhibits stable association with the 40 S ribosomal subunit and prevents translation [27]. Decay is mediated through binding of the zinc finger protein tristetraprolin (TTP) to the ARE, as shown with TTP-deficient mice which show elevated levels of TNF-α due to an increase in the mRNA half-life [30]. Additional proteins such as huR and TIA-1 interact with the ARE and influence mRNA stability and translation [31,32]. In addition the constitutive decay element (CDE) was described which constitutively mediates decay of TNF-α mRNA [25]. However no proteins were so far described which may bind to the CDE element.

Our experiments using RAW264.7 and RAW-IFIT-2 cells transfected with EGFP constructs flanked by different 3'UTRs clearly indicate that the characteristics of the 3'UTRs define whether forced IFIT-2 expression can mediate inhibition of protein expression or not. Deletion of parts of the 3'UTR of TNF-α revealed that the distal part of the 3'UTR containing the CDE element is sufficient for IFIT-2 mediated inhibition of protein expression while the ARE element has no impact. How IFIT-2 controls protein expression via 3'UTRs remains to be elucidated in further studies. Taken together, our data suggest that IFIT-2 acts as a regulatory protein which may repress the protein expression of distinct genes.

At this point, however, the number of proteins which may be affected by IFIT-2 is not known. Therefore, it is hard to predict how this function of IFIT-2 may translate finally into in vivo function of IFIT-2 as an interferon induced effector protein. In further studies it is necessary to investigate the in vivo function of IFIT-2 by additional tools such as IFIT-2 knock-out mice.

## Methods

### Reagents and antibodies

Ultra pure LPS from *S. enterica *serovar Minnesota was purchased from EMD Chemicals (Gibbstown, NJ). Recombinant mouse IFN-β and IFN-γ were purchased from R&D Systems (Minneapolis, MA). Actinomycin D was obtained from Sigma (St Louis, MO). Anti-mouse β-actin antibody was purchased from Sigma. Antibodies against phospho-p44/42, p44/42, IκB-α, phospho-IκB-α were from Cell Signaling Technology (Danvers, MA). Pan-JNK/SAPK1, phospho-JNK/SAPK, p38 and phospho-p38 specific antibodies were purchased from BD Biosciences (Franklin Lakes, NJ). Anti-EGR-1 antibody was from Santa Cruz (Santa Cruz, CA) and anti-Histone H3 from BioLegend (San Diego, CA). Horseradish peroxidase labeled rabbit anti-mouse and swine anti-rabbit antibodies were obtained from DAKO (Glostrup, Denmark). Monoclonal antibodies against the mouse IFN-α/β receptor subunit 1 (MAR-1) [33] and the human IFN-γR (GIR-218) [34] were kindly provided by R.D. Schreiber and Kathleen C.F. Sheehan, Washington University St. Louis.

### Cell culture and cell culture assays

RAW264.7 cells were maintained in Dulbecco's modified Eagle's medium (Invitrogen, Carlsbad, CA) supplemented with 10% fetal bovine serum (Sigma), 4 mM L-glutamine (Invitrogen), 1 mM sodium pyruvate (Biochrom KG, Berlin, Germany), 100 units/ml penicillin (Invitrogen) and 100 μg/ml streptomycin (Invitrogen) in a humidified 5% CO_2 _atmosphere at 37°C.

### Plasmids and preparation of stable transformants

The full-length mIFIT2 was amplified by PCR from cDNA of IFN-β stimulated RAW cells using the primers 5'-GGA TCC ATG AGT ACA ACG AGT AAG GAG TCA C3' and 5'-GCG GCC GCC TAG TAT TCA GCA CCT GCT TCA TCC-3'. The PCR fragment was cloned into pEF4/V5-His A (Invitrogen, Carlsbad, CA). The plasmid was delivered to RAW264.7 cells using ExGen transfection reagent (Fermentas, Burlington, Canada). Transfectants were selected in Zeocin™ containing medium (0.5 mg/ml) (Invitrogen).

pMCherry-C1 was generated by replacing the DsRed2 sequence of the expression vector pDsRed2-C1 (Invitrogen) by the sequence of mCherry. mCherry was generated by PCR using the vector mCherry-pRSET-B [35] as a template kindly provided by Roger Y. Tsien, University of San Diego. To obtain constructs which encode for EGFP and contain 3'UTRs of TNF-α, fragments of the 3'UTR of TNF-α, 3'UTR of MIP-2, IL-6, IFIT-1 and VEGF, cDNA from LPS stimulated RAW264.7 cells was used as template to amplify PCR products using specific oligonucleotides which contained different restriction sites (TNF: P1, 5'-CTC GAG TGA AGG GAA TGG GTG TTC ATC-3', 5'-GGA TCC CTT TTC CAA GCG ATC TTT ATT TCT CTC-3'; P3, 5'-AAG CTT TGA ATG TAT TTA TTT GGA AGG CCG-3', 5'-GGA TCC CTT TTC CAA GCG ATC TTT ATT TCT CTC-3'; P7 5'-CTC GAG TGA AGG GAA TGG GTG TTC ATC-3', 5'-GGA TCC GGG TCC TCC AGG ACA CCC C-3'; IFIT1, 5'-CTC GAG TGA ATG CAG CTC ACC TCT GTG-3', 5'-GGA TCC GAG GGG AAT ATG TTT ATT TGG AC-3'; IL-6 5'-CTC GAG TAG TGC GTT ATG CCT AAG CAT ATC AG-3', 5'-TTT ATT TGT TTG AAG ACA GTC TAA ACA TTA TAA AAA TAC ATC CA-3'; MIP-2, 5'-CTC GAG TGA CCT GGA AAG GAG GAG CC-3', 5'-GGA TCC AAC AGA CGT TTT TAT TTT TTG TTA TTT G-3'; VEGF 5'-CTC GAG TGA GCC AGG CTG CAG GAA GG-3', 5'-AAG CTT TTT GAG ATC AGA ATT CAA TTC TTT AAT AC-3'). PCR products were subcloned into pCR-Blunt II Topo and then cloned into pEGFP-C3. All constructs were verified by sequencing. Sequencing of the TNF-α 3'UTR which was also used as template for all TNF 3'UTR mutants revealed a slight modification at the 3' end in comparison to the reference sequence NM_013693 resulting at position 1601–1605 in AGTCA instead of GGGTC.

### Preparation of GST fusion proteins and antibody production

DNA fragments encoding mIFIT-2 and mIFIT-1 were subcloned into GST fusion protein expression vector pGEX-4T3 (Amersham Biosciences) and transformed into *E. coli *strain BL21. GST fusion proteins were expressed and purified by affinity chromatography. Anti-IFIT-1 and IFIT-2 antibodies were raised in rabbits by immunizing with a full-length protein of bacterially expressed recombinant GST-IFIT-1 and GST-IFIT-2. The generated antibodies were subsequently affinity-purified (Biogenes, Berlin, Germany).

### Western Blotting

2 × 10^6 ^RAW cells were seeded overnight in 6-well plates and then stimulated under different conditions. At different time points cells were washed with PBS and lysed for 30 min on ice in lysis buffer (50 mM Tris-HCl pH 7.5, 150 mM NaCl, 0.5% NP40, 400 μM DTT, 1 mM Na_3_VO_4_) containing a protease inhibitor cocktail (Roche Diagnostics, Mannheim, Germany). The insoluble material was removed by centrifugation at 15000 g for 10 min at 4°C. Protein concentration was determined using the Bio-Rad protein assay (Bio-Rad, Hercules, CA) according to the manufacturer's instructions. 25–50 μg of protein were separated on sodium dodecyl sulfate (SDS)-polyacrylamide gels and blotted onto Immobilon-P PVDF membranes (Millipore, Bedford, MA). The membranes were blocked with 5% nonfat dry milk in TBST (10 mM Tris-HCl, 150 mM NaCl, 0.5% Tween 20, pH 7.4) for 1 h at room temperature. Incubation with primary antibodies was performed overnight at 4°C followed by incubation with the appropriate HRP-conjugated secondary antibody for 1 h at room temperature. Immunoreactive bands were visualized using the ECL system (GE Healthcare, Köln, Germany) according to the manufacturer's instructions. In some cases band intensities of immunoblots were quantified and normalized to house keeping proteins by using Quantity One software, Bio-Rad Laboratories (Hercules, CA). Ratio of band intensities of RAW264.7 and RAW-IFIT-2 cell lysates was calculated for each immunoblot. Means and standard deviations presented were obtained from at least three experiments.

To generate nuclear extracts cells were scraped off in PBS and centrifuged for 5 min at 400 g. Cells were lysed in hypotonic buffer A (20 mM Hepes pH 7.9, 10 mM KCl, 0.1 mM Na_3_VO_4_, 1 mM EDTA, 0.2% NP40, 10% glycerol, 1 mM DTT, complete protease inhibitor cocktail (Roche)) for 5 min on ice. Nuclei were pelleted for 2 min at 15000 g and suspended in hypertonic buffer B (420 mM NaCl, 20% glycerol, 20 mM Hepes pH 7.9, 10 mM KCl, 0.1 mM Na_3_VO_4_, 1 mM EDTA, complete protease inhibitor cocktail, 1 mM DTT). After 30 min incubation on ice insoluble material was removed by centrifugation and nuclear extracts were stored at -80°C.

### Quantitative RT-PCR analysis

Total RNA of RAW264.7 cells seeded in 6-well plates at a density of 2 × 10^6 ^cells per well was extracted using the RNeasy Mini Kit (Qiagen). 2 μg of RNA were reverse transcribed as described [36]. Semiquantitative Real-time PCR was performed on a TaqMan 5700 (Applied Biosystems, Foster City, CA), using Platinum qPCR Super Mix UDG (Invitrogen) and the Assay-on-Demand primer/probe mixes Mm00657299_g1 (ribosomal protein L8 (RPL8), Mm00515153_m1 (IFIT-1) and Mm00443258_m1 (TNF-α) (Applied Biosystems, Foster City, CA). Results were quantified using the 2^-ΔΔC^_T _method [37,38]. TNF-α and IFIT-1 mRNA expression levels were normalized to the expression of the house keeping gene RPL8.

### Measurement of cytokines

3.5 × 10^5 ^RAW cells were seeded overnight in 24 well plates and then stimulated under different conditions. At different time points supernatants were collected. ELISAs were performed to measure concentrations of TNF-α and IL-6 (BD Biosciences), VEGF and MIP-2 (R&D systems) according to the manufacturer's instructions.

### Detection of IFIT-2 expression in cells

For immune fluorescence staining, cells were cultured on glass slides in 24 well plates. After fixation of the monolayers with 3% paraformaldehyde solution and subsequent permeabilization with 2% Triton-X100 in PBS, cells were stained with rabbit polyclonal anti-mouse IFIT-2 antibody. Subsequently cells were stained with a goat anti-rabbit Cy2 (Dianova, Hamburg, Germany) and TRITC-conjugated phalloidin (Sigma) followed by DAPI (Merck, Darmstadt, Germany). The fluorescence images were obtained with a confocal laser scanning microscope (Leica DM IRE2). Intracellular IFIT-2 staining was performed using the Cytofix/Cytoperm kit (BD Biosciences) according to the manufacturer's instructions. For staining a polyclonal anti-IFIT-2 and goat-anti-rabbit IgG-PE antibody (BD Biosciences) were used.

### Determination of EGFP and mCherry expression by flow cytometry

The expression vector pmCherry-C1 was delivered into RAW cells by nucleofection using an Amaxa electroporator (Amaxa, Gaithersburg, MD) according to the manufacturer's instructions. Cells were co-transfected with pEGFP-C3 and pEGFP-C3 variants encoding different 3'UTRs as illustrated in Figure 7 and 8 and mutants of the 3'UTR of TNF-α. Four hours after transfection cells were harvested and analyzed by flow cytometry using a FACSCalibur (Becton Dickinson, Heidelberg, Germany). To determine changes in EGFP expression between RAW264.7 and RAW-IFIT-2 cells only living cells expressing mCherry were included for histogram analysis of EGFP expression.

### Statistics

Statistical analyses were performed using the Graph pad prism 4.0 software and the appropriate statistical test as indicated in the figures. For statistical analysis of cytokine secretion determined by ELISA we used the means and standard deviations of four samples, representing two biological replicates and from each biological two technical replicates. For mRNA expression the means and standard deviations of two biological replicates were analyzed. All experiments were repeated in total three times if not otherwise stated.

## Authors' contributions

SB participated in the design of the study, carried out most of the experiments, performed data analyses and helped to draft the mansucript. JK, JG and BM carried out some of the experiments and were involved in data analyses. IBA participated conceptually and in the draft of the manuscript. EB conceived the study, carried out the design and coordination and drafted the manuscript.
